# Is the Superficial Peritendinous Tissue an Additional Pain Driver in Patellar Tendinopathy?—Studies on Morphology and Innervation in a Case Series

**DOI:** 10.3390/medicina58050601

**Published:** 2022-04-27

**Authors:** Christoph Spang, Lorenzo Masci, Håkan Alfredson

**Affiliations:** 1 Department of Integrative Medical Biology, Anatomy Section, Umeå University, 901 87 Umeå, Sweden; 2 Private Orthopaedic Spine Center, 97080 Würzburg, Germany; 3 Institute of Sports Exercise and Health, University College Hospital London, London W1T 7HA, UK; lorenzo@sportdoctorlondon.com; 4 Sports & Exercise Medicine, Queen Mary University of London, London E1 4DG, UK; 5 Department of Community Research and Rehabilitation, Sports Medicine, Umeå University, 901 87 Umeå, Sweden

**Keywords:** patellar tendinopathy, innervation, surgical treatment, peritendinous tissue

## Abstract

*Background and Objectives*: Ultrasound (US) and Doppler (DP) guided arthroscopic shaving targeting the area with neovessels and nerves on the dorsal side of the tendon has shown good clinical results. Recently, we observed that in a sub-group of patients there is also local tenderness on the superficial side of the proximal patellar tendon; *Material and Methods*: The aim was to examine morphology and innervation patterns of the superficial peritendinous tissue from patients (four men and two women; mean age 23 years, range 17–31 years) that on US+DP examination showed a locally thickened paratenon including high blood flow. Tissue sections were stained for morphology (hematoxylin and eosin, H&E) and immunohistochemically for nerve markers (β-tubulin; tyrosine hydroxylase, TH; calcitonin related gene peptide, CRGP); *Results:* All tissue specimens contained high levels of blood vessels and nerves (fascicles, sprouting nerve fibers, perivascular innervation) as evidenced by evaluation for H&E and β-tubulin reactions. Nerve fascicles mainly contained sensory but also sympathetic axons. Nerves related to blood vessels were sympathetic fibers; *Conclusions:* There was a marked innervation in the superficial peritendinous tissue in a sub-group of patients with patellar tendinopathy and severe tenderness in the proximal patellar tendon. The results indicate that this tissue might be an additional pain driver in some patients and should be considered in further studies.

## 1. Introduction

Patellar tendinopathy is known to be a troublesome condition to treat [1,2]. When conservative treatments fail, surgery is indicated, and the traditional surgical treatment method is intra-tendinous revision surgery. However, the results with this tendon invasive method have been shown to be poor, with only 50% good clinical results [2].

A new surgical treatment method, ultrasound-guided arthroscopic shaving, has shown good and reliable clinical results deleted word in clinical studies [3,4,5,6]. This method is based on the findings from ultrasound and Doppler-guided biopsies, showing that nerves were located outside the deep side of the tendon [7]. The ultrasound-guided surgical treatment is targeting the regions with nerves outside the tendon, and no intra-tendinous revision surgery is performed. This allows for a more aggressive rehabilitation and a faster return to full tendon loading sport activities [4,5,6].

In a sub-group of patients with patellar tendinopathy, athletes subjected to direct trauma to the anterior side of the patellar tendon, such as landing on a bent knee, we have observed a very distinct and disabling local tenderness on the superficial side of the proximal patellar tendon. In these patients, ultrasound and Doppler examination shows a localized thickening of the paratenon, including high blood flow, on the superficial side of the proximal patellar tendon. Ultrasound (US) and Doppler (DP)-guided arthroscopic shaving and additional open scraping of this superficial peritendinous tissue have resulted in good clinical outcomes [6]. 

The aim with this study was to characterize this richly vascularized locally thickened paratenon, in a deleted word sub-group of patients with patellar tendinopathy by studying morphology and innervation patterns.

## 2. Materials and Methods

### 2.1. Patients and Clinical Procedures

The study was performed at the Department of Anatomy, Umeå University, Umeå, Sweden. Ethical approval to study tendon tissue samples was achieved from the Ethical Board Umeå University.

**Figure 1:**
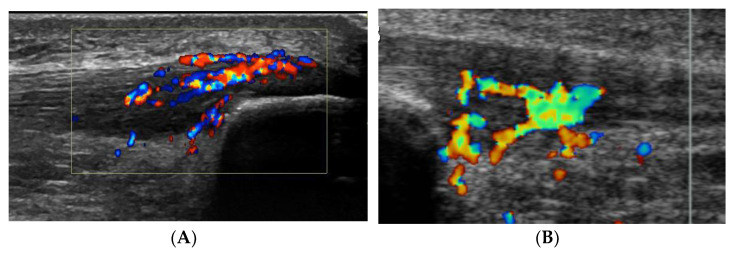
(**A**,**B**). Ultrasound and Doppler picture from 2 patients suffering from chronic painful patellar tendinopathy. There is a thickened proximal patellar tendon, including irregular tendon structure, hypo-echoic regions and high blood flow on the dorsal side of the tendon. (**A**): This patient also had severe tenderness on the superficial side of the proximal tendon, where there was a localized thickening of the paratenon including high blood flow. (**B**): This patient had no tenderness on the superficial side of the proximal tendon, where there was a normal paratenon and normal blood flow.

Six patients (4 men and 2 women; mean age 23 years, range 17–31 years) were included. All patients were sports active and had suffered from ultrasound and Doppler verified patellar tendinopathy in the proximal part of the patellar tendon for more than 3 months [5]. In all deleted word these patients, there was also severe tenderness localized on the superficial side of the proximal patellar tendon, and ultrasound+Doppler examination showed a thickened paratenon including high blood flow in that region (Figure 1A). 

**Figure 2:**
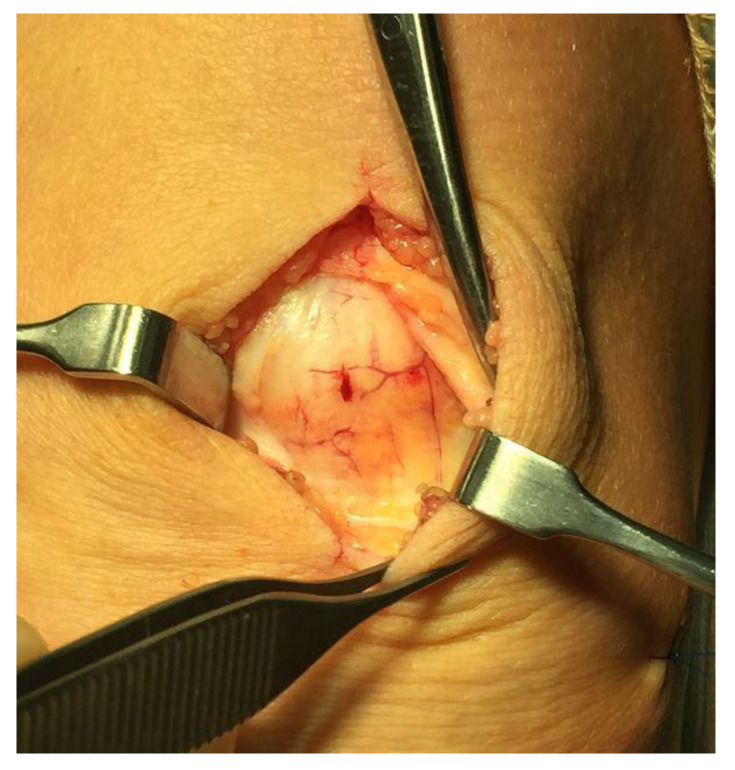
Surgical inspection of the superficial peritendinous tissue. Richly vascularized fatty tissue can be observed.

All patients underwent deleted words ultrasound (US) and Doppler (DP)-guided arthroscopic shaving and also open superficial scraping deleted words (Figure 2) [6]. Tissue sections from the superficial peritendinous tissue of the patellar tendon were analyzed for morphology and innervation patterns. 

### 2.2. Sampling, Fixation and Sectioning

Immediately after surgery, the tissue specimens were put in fixative solution (4% formaldehyde in 0.1 M phosphate buffer, pH 7.0) at 4 °C overnight. Then the samples were washed three times in Tyrode’s solution containing 10% sucrose (pH 7.2), the first washing step being performed at 4 °C overnight. Before freezing, samples were divided into smaller pieces and then placed on a thin cardboard surrounded by OCT embedding medium (TissueTek, Miles Laboratories, Naperville, IL, USA). Eventually, the cardboard with the specimen was dipped in liquid propane chilled with liquid nitrogen and then stored at −80 °C until use. For immunohistochemical analyses, specimens were cryosectioned with a thickness of 7 μm (Leica Microsystem CM 300, Heidelberg, Germany) and mounted on superfrost plus slides (Thermo Scientific, Braunschweig, Germany).

### 2.3. Immunohistochemistry

Parallel sections were stained for morphology (hematoxylin and eosin (H&E) staining) [8] and immunohistochemically for markers for general nerve (axonal) fibers βIII-tubulin (Sigma-Aldrich, St. Louis, MO, USA; code: T8660; mouse monoclonal), sympathetic nerve fibers (tyrosine hydroxylase, TH; Pel-Freez Arkansas; code: P40101; rabbit polyclonal) and sensory nerve fibers (calcitonin related gene peptide, CRGP; Santa Cruz Biotechnologies, code: sc-8856; goat polyclonal). The staining procedure was performed according to an established, previously described, protocol [9,10].

For staining for monoclonals (TH), rabbit normal serum (code X0902, DakoCytomation) and TRITC-conjugated rabbit anti-mouse (code R0276, DakoCytomation) was applied. Donkey normal serum (code: 017-000-121; Jackson Immune Research Laboratories Inc., West Grove, PA, USA) and FITC-conjugated donkey anti-goat secondary antibody (code: 705-095-147; Jackson Immune Research Inc.) were used for stainings for CGRP. For sections stained for TH, swine normal serum (code: 014-000-121; Jackson Immune Research Inc.) and TRITC-conjugated swine anti-rabbit secondary antibody (code: R0156, DakoCytomation) were used. Furthermore, normal serum and primary and secondary antibodies were diluted in 0.1% bovine serum albumin (BSA) in 0.01 M PBS (pH 7.4). When using goat antibodies (CGRP), all dilutions were made without BSA.

For all antibodies, control stainings using PBS instead of primary antibody were performed. The specificity of these antibodies in human tendon tissue has been shown in previous studies [9,10]. The microscopical evaluation was carried out using a Zeiss Axioscope 2 plus microscope equipped with epifluorescent technique and an Olympus DP70 digital camera. Figure montages were created using Adobe Photoshop CS5.

## 3. Results

**Figure 3:**
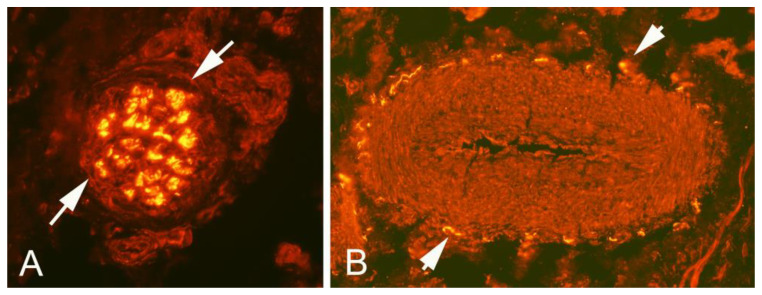
Sections of superficial side peritendinous tissue from the patellar tendon stained for general nerve (axonal) marker bIII tubulin. Nerve fibers were seen in nerve fascicles ((**A**), arrows) and in close vicinity of blood vessels and perivascular spaces ((**B**), arrow heads).

**Figure 4:**
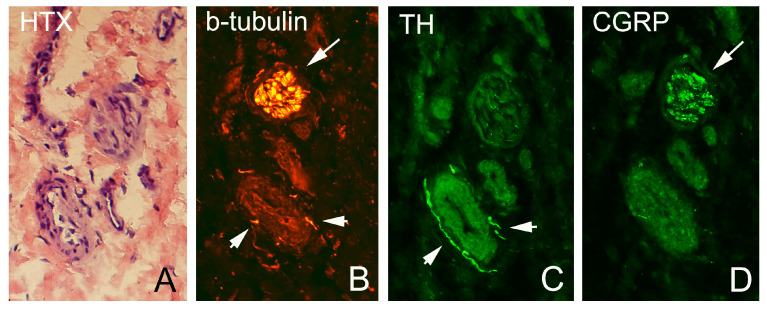
Parallel sections of superficial peritendinous tissue of the patellar tendon stained for HTX (**A**), bIII-tubulin (**B**), TH (**C**) and CGRP (**D**). TH immunoreactions are most pronounced in close vicinity to blood vessels and in perivascular spaces (arrow heads, (**C**)), CGRP immunoreactions are most pronounced in nerve fascicles (arrows, (**D**)).

**Figure 5:**
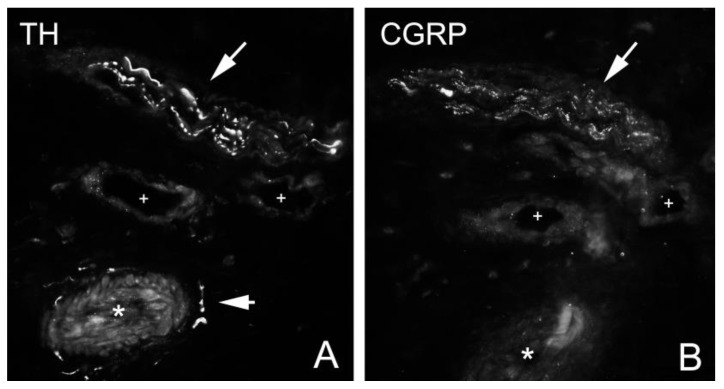
Parallel sections stained for TH (**A**) and CGRP (**B**). The nerve fascicle contains positive immunoreactions both for TH and CGRP (arrows). Perivascular immunoreactions were only seen for TH (arrow head). Asterisks and crosses indicate the position of the same blood vessel.

As described above, all ultrasound+Doppler examinations showed a thickened para-tenon including high blood flow in the superficial peritendinous region (Figure 1A). This was in contrast to the majority of patients with patellar tendinopathy where there is a normal paratenon and normal blood flow (Figure 1B). All tissue specimens contained large portions of loose connective tissue with high numbers of blood vessels and also fat tissue. There were multiple nerve fascicles and sprouting nerve fibers as evidenced by evaluation for HTX and β-tubulin reactions (Figure 3 and Figure 4). Often those nerves were found in close vicinity to blood vessels and perivascular in blood vessel walls (Figure 3). Sensory innervation (CGRP positive nerve fibers) was mostly found in axons of nerve fascicles (Figure 4 and Figure 5). Sympathetic innervation (TH positive nerve fibers) was found both in nerve fascicles and perivascular in big and small blood vessels.

## 4. Discussion

This study showed that there was a marked sympathetic and sensory innervation in the superficial peritendinous tissue in a sub-group of patients with patellar tendinopathy and additional severe tenderness on the superficial side of the proximal patellar tendon. Ultrasound and Doppler examination showed in all these patients a thickened paratenon including high blood flow. 

The proximal patellar tendon in patients with chronic painful patellar tendinopathy has been scientifically evaluated, and traditionally the tendon changes are located dorsal and central in the tendon. Biopsies have shown that the nerves are located outside the dorsal side of the tendon [7]. These new findings show that in a sub-group of patients there are sensory nerves also on the superficial side which highlights this tissue as a potential pain driver. All patients in the current study were involved in sports or recreational activities where falls with landing on bent knee were common. Multiple traumas to the superficial side of the tendon are likely causing these changes, but that needs to be further studied. Theoretically, it could also be part of the tendinopathy changes. Consequently, in a sub-group of patients with patellar tendinopathy pain might be coming from both the traditional dorsal side and the superficial side of the proximal patellar tendon. The findings are of clinical importance when trying to relieve pain from the patellar tendon. Our experience with patients suffering from patellar tendinopathy and having this type of change is that if only the dorsal side is focused for treatment (via the ultrasound and Doppler-guided shaving procedure), there are in some patients after surgery remaining severe tenderness on the superficial side. However, if the superficial side changes are also treated (via an open scraping procedure), there is a better pain relief. Of course, these observations need to be further studied in clinical studies.

A weakness in the current study is that the morphological results are descriptive, and that the sample size is relatively small. The findings need to be verified also in a larger material. In addition, normal control tissue needs to be evaluated. However, despite these weaknesses, we believe the findings add new information by highlighting the superficial peritendinous tissue as a potential pain driver in a sub-group of patients with deleted word patellar tendinopathy and also superficial tenderness. 

## 5. Conclusions

In conclusion, for patients with chronic painful patellar tendinopathy, in a sub-group of patients suffering from severe tenderness also on the superficial side of the proximal patellar tendon, there are sensory nerves not only on the dorsal side but also on the superficial side of the tendon. The findings can be of importance for pain treatment in patients with chronic painful patellar tendinopathy. 

## Data Availability

The data presented in this study are available on request from the corresponding author.

## References

[1] Khan K.M., Maffuli N., Coleman B.D., Cook J.L., Taunton J.E. (1998). Patellar tendinopathy: Some aspects of basic science and clinical management. Br. J. Sports Med..

[2] Bahr R., Fossan B., Løken S., Engebretsen L. (2006). Surgical treatment compared with eccentric training for patellar tendinopathy (Jumpers knee): A randomized controlled trial. J. Bone Jt. Surg. Am..

[3] Willberg L., Sunding K., Ohberg L., Forssblad M., Alfredson H. (2007). Treatment of Jumper’s knee: Promising short-term results in a pilot study using a new arthroscopic approach based on imaging findings. Knee Surg. Sports Traumatol. Arthrosc..

[4] Willberg L., Sunding K., Forssblad M., Fahlstrom M., Alfredson H. (2011). Sclerosing polidocanol injections or arthroscopic shaving to treat patellar tendinopathy/jumper’s knee? A randomised controlled study. Br. J. Sports Med..

[5] Sunding K., Willberg L., Werner S., Alfredson H., Forssblad M., Fahlstrom M. (2015). Sclerosing injections and ultrasound-guided arthroscopic shaving for patellar tendinopathy—good clinical results and decreased tendon thickness after surgery—A medium term follow-up study. Knee Surg. Sports Traumatol. Arthrosc..

[6] Alfredson H., Masci L.M. (2015). Ultrasound and Doppler-guided surgery for the treatment of Jumper’s knee in professional rugby players. PST Pain Stud. Treat..

[7] Danielson P., Andersson G., Alfredson H., Forsgren S. (2008). Marked sympathetic component in the perivascular innervation of the dorsal paratendinous tissue of the patellar tendon in arthroscopically treated tendinosis patients. Knee Surg. Sports Traumatol. Arthrosc..

[8] Spang C., Scott A., Danielson P., Lorentzon R., Forsgren S. (2012). VGluT2 and NMDAR1 expression in cells in the inflammatory infiltrates in experimentally induced myositis: Evidence of local glutamate signaling suggests autocrine/paracrine effects in an overuse injury model. Inflammation.

[9] Spang C., Alfredson H. (2017). Richly innervated soft tissues covering the superficial aspect of the extensor origin in patients with chronic painful tennis elbow—Implication for treatment?. J. Musculoskelet. Neuronal Interact..

[10] Spang C., Renström L., Alfredson H., Forsgren S. (2017). Marked expression of TNF receptors in human peritendinous tissues including in nerve fascicles with axonal damage—Studies on tendinopathy and tennis elbow. J. Musculoskelet. Neuronal Interact..

